# MALDI-TOF MS Applications to the Detection of Antifungal Resistance: State of the Art and Future Perspectives

**DOI:** 10.3389/fmicb.2018.02577

**Published:** 2018-10-30

**Authors:** Walter Florio, Arianna Tavanti, Emilia Ghelardi, Antonella Lupetti

**Affiliations:** ^1^Dipartimento di Ricerca Traslazionale e delle Nuove Tecnologie in Medicina e Chirurgia, Università di Pisa, Pisa, Italy; ^2^Dipartimento di Biologia, Università di Pisa, Pisa, Italy

**Keywords:** MALDI-TOF mass spectrometry, antimicrobial resistance, antifungal susceptibility testing, blood culture, rapid AFST

## Abstract

MALDI-TOF MS technology has made possible revolutionary advances in the diagnosis of infectious diseases. Besides allowing rapid and reliable identification of bacteria and fungi, this technology has been recently applied to the detection of antimicrobial resistance. Several approaches have been proposed and evaluated for application of MALDI-TOF MS to antimicrobial susceptibility testing of bacteria, and some of these have been or might be applied also to yeasts. In this context, the comparison of proteomic profiles of bacteria/yeasts incubated with or without antimicrobial drugs is a very promising method. Another recently proposed MALDI-TOF MS-based approach for antifungal susceptibility testing is the application of the semi-quantitative MALDI Biotyper antibiotic susceptibility test rapid assay, which was originally designed for antimicrobial susceptibility testing of bacteria, to yeast isolates. Increasingly effective and accurate MS tools and instruments as well as the possibility to optimize analytical parameter settings for targeted applications have generated an expanding area in the field of clinical microbiology diagnostics, paving the way for the development and/or optimization of rapid methods for antifungal susceptibility testing in the near future. In the present study, the state of the art of MALDI-TOF MS applications to antifungal susceptibility testing is reviewed, and cutting-edge developments are discussed, with a particular focus on methods allowing rapid detection of drug resistance in pathogenic fungi causing systemic mycoses.

## Introduction

During the last two decades, MALDI-TOF MS technology has rapidly evolved toward new applications in the field of clinical diagnostic microbiology, generating a novel, robust and accurate tool for rapid identification of bacteria and yeasts (Kliem and Sauer, 2012; Richter et al., 2012; Spanu et al., 2012; Szabados et al., 2012; Idelevich et al., 2014; Barnini et al., 2015; Florio et al., 2018b). In parallel, major efforts have been made to expand and apply the enormous potentialities of this technology to another important issue: testing resistance and, in some cases, susceptibility of microorganisms to antimicrobial drugs. The emergence and diffusion of bacterial and fungal pathogens resistant to multiple drugs and the high incidence of hospital-acquired bloodstream infections (BSIs) have made the purpose of developing new methods for rapid and reliable antimicrobial susceptibility testing an urgent issue. In this context, considerable efforts have been made in order to apply MALDI-TOF MS technology to the detection of antimicrobial resistance, especially for multi-drug resistant (MDR) microorganisms causing systemic infections (Nordmann et al., 2011; Cantòn et al., 2012; Patel and Bonomo, 2013; Lamoth et al., 2018), which represent a major concern for public health (Walsh, 2010; Walsh and Gamaletsou, 2013; Lee et al., 2016; Barchiesi et al., 2017). Several MALDI-TOF MS-based approaches have been attempted for the detection of antibiotic resistance in bacteria. These include: (i) the assessment of β-lactamase activity by MALDI-TOF MS detection of hydrolysis products of β-lactam/carbapenem antibiotics (Hooff et al., 2012; Hrabák et al., 2012; Jung et al., 2014; Oviano et al., 2016); (ii) identification in mass spectra of genes (Lau et al., 2014; Rodríguez-Baño et al., 2018) or peptides specifically involved in antibiotic resistance mechanisms (Gaibani et al., 2016; Chang et al., 2018); (iii) identification of biomarkers somehow correlated with, though not responsible for drug resistance, such as the phenol-soluble protein toxin (PSM-mec), produced by a subset of methicillin-resistant *Staphylococcus aureus* strains (Chatterjee et al., 2011), and detectable by MALDI-TOF as a 2415 ± 2.00 m/z peak (Rhoads et al., 2016); and (iv) assays based on discrimination of mass spectra of resistant from susceptible microbial isolates after exposure to breakpoint concentrations of antimicrobial agents directly on the target plate for MALDI-TOF MS analysis (Idelevich et al., 2017). This latter approach seems very promising since, theoretically, it might be applied to any microbial species and antimicrobial agent independently from the underlying resistance mechanisms, and it may be suitable for laboratory automation and simultaneous testing of a panel of different antimicrobial drugs.

As multi-drug resistance is an increasing widespread problem also in fungal infections, there is a pressing need for rapid methods allowing to obtain timely and reliable information on antifungal susceptibility/resistance of fungal infectious agents, especially those causing systemic mycoses. In spite of advances in diagnosis and treatment, the incidence of invasive fungal infections has dramatically increased during the last two decades (Barchiesi et al., 2016; Benedict et al., 2017; Cornely et al., 2017; Guo et al., 2017; Pana et al., 2017), with only a few classes of antifungal drugs being available (Pfaller, 2012; Pierce and Lopez-Ribot, 2013; Patil and Majumdar, 2017). The growing elderly population, frequently suffering from co-morbidities, high colonization rate by *Candida albicans*, immunosuppressive treatment after organ transplantation, and prolonged antibiotic therapies have substantially contributed to such an increase in fungal infections (Guimarães et al., 2012; Wang et al., 2014; Barchiesi et al., 2017). Although *C. albicans* remains the most frequently isolated species, other fungal pathogens have been isolated with remarkably increasing frequency; these include *Candida glabrata* (Cleveland et al., 2012; Pfaller et al., 2014), the emerging pathogen *Candida auris* (Bao et al., 2018; Kohlenberg et al., 2018; Kordalewska et al., 2018), *Trichosporon* spp. (Davies and Thornton, 2014), and filamentous fungi, such as *Scedosporium* spp., mucoralean fungi, and *Fusarium* spp. (Walsh and Gamaletsou, 2013; Davuodi et al., 2015).

Systemic infections sustained by *Candida* spp. as well as by other fungal pathogens are associated with mortality rates that can be higher than 60%, depending on the patient category (Kett et al., 2011; Kollef et al., 2012; Barchiesi et al., 2016, 2017; Guo et al., 2017). Since timely administration of effective antifungal therapy is of vital importance for the outcome of patients affected by these infections (Garey et al., 2006) and susceptibility profiles to antifungal agents vary greatly among fungi, rapid species identification and antifungal susceptibility testing (AFST) is fundamental to reduce mortality and improve patients’ outcome. MALDI-TOF MS, extensively used for identification of bacteria, has been increasingly proposed also for rapid identification of fungal pathogens directly in positive BCs (Ferreira et al., 2011; Yan et al., 2011; Spanu et al., 2012; Idelevich et al., 2014; Vecchione et al., 2018). Yeast identification by itself provides relevant clinical information since different fungal species may differ in virulence and drug resistance. For example, the antimicrobial susceptibility profile of *Candida parapsilosis* and *C. glabrata* can be quite different, with *C. parapsilosis* more frequently resistant to echinocandins and *C. glabrata* to azoles (Silva et al., 2012). Furthermore, invasive trichosporonosis is characterized by resistance to amphotericin and echinocandins, and poor prognosis (Miceli et al., 2011). Therefore, the ability to rapidly identify these yeasts may be useful to promptly streamline empirical antimicrobial therapy. However, the emergence and spread of MDR fungal pathogens (Lamoth et al., 2018) have posed a pressing need for rapid AFST. In general, echinocandins are widely used as empirical antifungal therapy for patients with candidemia, at least until AFST results become available; when resistance is detected, treatment with echinocandins needs to be immediately streamlined. Therefore, a rapid method for AFST may be of vital importance to achieve a favorable outcome, especially in critically ill patients. To this purpose, the application of MALDI-TOF MS to AFST has been attempted in the last few years (Vella et al., 2013; Saracli et al., 2015; Vatanshenassan et al., 2018). In this context, two methods have been proposed, which revealed potential for the development of rapid assays based on MALDI-TOF MS. The first is based on the comparative analysis of mass spectra of fungal isolates exposed to different concentrations of antifungal drugs (Vella et al., 2013, 2017; Saracli et al., 2015). The second relies on the application and optimization of the MALDI Biotyper antibiotic susceptibility test rapid assay (MBT ASTRA), originally developed for rapid antibiotic susceptibility testing in bacteria (Sparbier et al., 2016), to AFST (Vatanshenassan et al., 2018).

The present review provides a synthetic, updated overview of the proposed methods based on MALDI-TOF MS and aimed at yielding rapid and accurate information regarding antimicrobial resistance of clinically relevant fungi.

## Antifungal Susceptibility Testing by Comparison of Composite Correlation Indexes

A method has been proposed for the detection of resistance to specific antifungal agents that relies on the comparison of MALDI-TOF MS spectra of yeast strains after incubation with high, intermediate or null antifungal concentrations (De Carolis et al., 2012; Vella et al., 2013, 2017; Saracli et al., 2015; Sanguinetti and Posteraro, 2016). In particular, MALDI-TOF MS spectra of yeast isolates after exposure to high, intermediate or null drug concentrations are used to generate a composite correlation index (CCI). The intermediate drug concentration is the minimal concentration of the compound able to induce a detectable proteome change in the mass spectrum of a susceptible strain. When a susceptible strain is tested, the mass spectrum at the intermediate drug concentration is more similar to that at the high concentration; therefore, the CCI of the intermediate/null concentration is lower than that of the intermediate/high concentration. This method has been used to test the resistance of *C. albicans, C. tropicalis*, and *C. glabrata* to triazole drugs. The results showed essential agreement ranging between 54 and 97% for the MALDI-TOF method in comparison to conventional AFST. The wide variability in essential agreement was dependent on both the triazole drug and *Candida* species tested (Saracli et al., 2015). A limit of this method was that the time saving over the conventional AFST method was of modest entity (overnight versus 24 h).

In another study based on the same approach (Vella et al., 2013), resistance to caspofungin could be detected in 10/11 (90.6%) *C. albicans* clinical isolates after an incubation of only 3 h, and 51/51 (100%) isolates were correctly classified as susceptible. The single very major error (false susceptibility) related to an isolate with a low level of caspofungin resistance. Caspofungin selectively targets the fungal cell wall by inhibiting 1,3-β-glucan synthase. Due to its high efficacy and reduced cases of side effects and adverse events in comparison with other antifungal agents, caspofungin has become a front-line agent in the treatment of candidemia and other invasive fungal infections (Patil and Majumdar, 2017). However, resistance to caspofungin can arise because of mutations in the *FSK1* gene in *C. albicans* (Balashov et al., 2006), and *FSK1* and *FSK2* genes in *C. glabrata* (Pham et al., 2014). Due to the emergence of drug resistant yeast strains (Chen et al., 2011), rapid detection of caspofungin resistance in *Candida* spp. clinical isolates is essential for timely and appropriate treatment of systemic fungal infections.

Recently, the same method based on detection of changes in the protein spectrum after a 3-h incubation with antifungal drugs was used to test a panel of 80 *C. glabrata* clinical isolates against anidulafungin or fluconazole (Vella et al., 2017). In comparison to the reference method recommended by the Clinical and Laboratory Standards Institute [CLSI], 2012), 58/58 (100%) susceptible isolates were classified as susceptible, and 11/22 (50.0%) isolates in the resistant category were classified as resistant to anidulafungin. For fluconazole, 40/41 (97.6%) susceptible isolates were classified as susceptible, and 37/39 (94.9%) isolates in the resistant category were classified as resistant. Of interest, when the assay was repeated for the 11 resistant isolates giving very major error with longer incubation times (6, 9, and 12 h), only two errors for anidulafungin remained unresolved; with a 15-h incubation, 100% essential agreement was reached with both the antifungal drugs. Therefore, incubation time was a critical factor to achieve maximum accuracy and reliability of this assay, especially for anidulafungin.

In a recent study (Paul et al., 2018), an assay based on CCI analysis was used to evaluate fluconazole resistance in 15 fluconazole resistant (MICs ranging from 16 to 128 μg/mL) and 19 fluconazole susceptible *C. tropicalis* isolates (MIC ≤ 1 μg/mL) in comparison with the reference CLSI microdilution method (Clinical and Laboratory Standards Institute [CLSI], 2012). In India, *C. tropicalis* is the commonest agent causing candidemia, and fluconazole resistant strains have been increasingly isolated (Chakrabarti et al., 2015). Therefore, a rapid method allowing the detection of fluconazole resistance in *C. tropicalis* strains grown in BCs may be very helpful to promptly streamline antifungal therapy. The authors reported that spectral changes were detectable by visual inspection soon after 4-h exposure to high (128 μg/mL), intermediate (1 μg/mL) or null concentrations of fluconazole for all *C. tropicalis* isolates. For software-based analysis, the incubation time was optimized at 5 h with 4 μg/mL as intermediate drug concentration. Among the 34 isolates tested, the minimal profile change concentrations (MPCCs), i.e., the minimum drug concentration at which an alteration of mass spectra can be detected, coincided with the MICs for 16 isolates (4 resistant, 12 susceptible), whereas the MPCC was one twofold dilution lower than the corresponding MIC in the remaining 18 isolates (11 resistant, 7 susceptible). Categorical and essential agreement were observed for all 34 isolates, indicating that CCI analysis might be used as a rapid screening method for fluconazole resistance in *C. tropicalis* clinical isolates.

## Detection of Caspofungin Resistance in *C. albicans* and *C. glabrata* by MBT ASTRA

In a recent proof of concept study (Vatanshenassan et al., 2018) the detection of *C. albicans* and *C. glabrata* strains resistant to caspofungin was evaluated by MBT ASTRA. This is a MALDI-TOF-based semi-quantitative technique, which was originally designed for rapid antibiotic susceptibility testing in bacteria. MBT ASTRA is a phenotypic assay comparing growth of microorganisms after incubation in the absence or in the presence of different concentrations of antimicrobial drugs. Cell growth is inferred from the comparison of the area under the curve (AUC) of the MALDI-TOF MS spectra for the different incubation setup (with or without antimicrobial drug). The relative growth (RG) is calculated for each concentration of antimicrobial agent as the ratio of the AUC observed with or without drug exposure, and a RG cutoff discriminating resistance from susceptibility is determined experimentally: strains showing RG above this cutoff are considered resistant, whereas those below the cutoff are considered susceptible. The MBT ASTRA results were compared with those obtained by the CLSI reference microdilution method (Clinical and Laboratory Standards Institute [CLSI], 2017) on 58 *C. albicans* and 57 *C. glabrata* clinical isolates. A categorical agreement of 100% was observed for 29 susceptible and 22 resistant strains of *C. albicans*. For seven *C. albicans* strains, insufficient growth was observed in the control setup (no caspofungin) and, therefore, these strains were excluded from further evaluation, resulting in a validity of the MBT ASTRA assay of 88%. For *C. glabrata*, 31 out of 33 strains categorized as resistant by the microdilution method resulted resistant also by MBT ASTRA, and four out of five susceptible strains were correctly detected. Eighteen out of 19 strains categorized as intermediate by the microdilution method resulted resistant by MBT ASTRA, while only one intermediate strain resulted susceptible. Therefore, a sensitivity of 94% and a specificity of 80% were observed using MBT ASTRA on *C. glabrata* strains. These results were obtained after a 6-h incubation of yeast cells with or without caspofungin. Time saving compared to the reference microdilution method was approximately 18 and 42 h for *C. albicans* and *C. glabrata*, respectively. The RG cutoff was set at 0.6 for both *C. albicans* and *C. glabrata*; breakpoint concentrations of 1 and 0.5 μg/mL caspofungin, discriminating susceptible from resistant strains, were determined for *C. albicans* and *C. glabrata*, respectively.

Overall, this approach seems promising, and it would be interesting to evaluate its performance in clinical settings, and the possibility to extend its application to the detection of resistance to other antifungal agents. In addition, it would be of great usefulness if it could be efficiently applied to yeasts directly recovered from positive BCs by lysis/centrifugation in Serum Separator Tubes (BD Vacutainer system) or using other suitable methods (Florio et al., 2018a). To this purpose, further studies might be necessary to optimize the analysis software and experimental conditions.

## Conclusion

**FIGURE 1:**
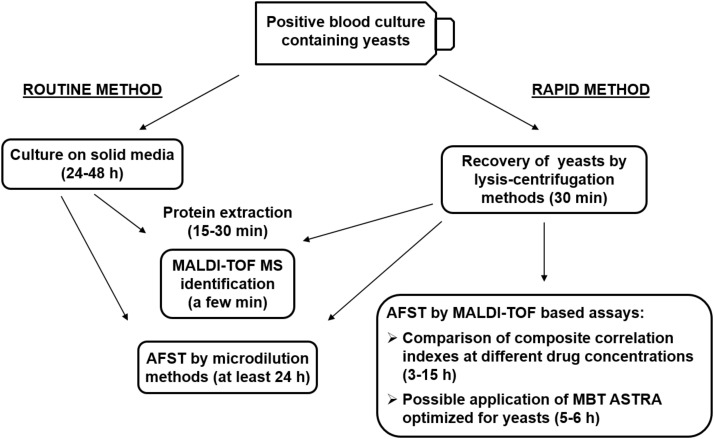
Schematic representation of the routine and rapid methods for identification and antifungal susceptibility testing (AFST) of yeasts grown in monomicrobial blood cultures, and possible applications of newly described methods. MBT ASTRA, MALDI Biotyper antibiotic susceptibility test rapid assay.

In this review, we summarized recently proposed methods and significant advances for rapid detection of antifungal resistance by MALDI-TOF MS with a particular focus on methods applicable to positive BCs, as schematically represented in Figure 1. The diagnosis of fungal BSIs by conventional methods is particularly time consuming, while in case of severe, life-threatening infections, such as systemic mycoses, timeliness and accuracy of test results are crucial factors for clinicians to decide and promptly administer an effective and targeted antifungal therapy. Conventional microdilution methods for AFST are generally very accurate but require long incubation times. Since mortality rates in systemic infections are strictly correlated with time between onset of symptoms and administration of effective therapy, rapid methods for AFST are strongly needed. Therefore, major research efforts have to be made to refine and optimize MALDI-TOF MS-based assays in order to obtain timely, accurate and reliable results. Because of the increasing diffusion of MDR fungal pathogens, methods that allow simultaneous susceptibility testing to different classes of antifungals are expected to represent a major focus of research in this field. Differently from bacteria, the existence of specific peaks associated with antimicrobial resistance in mass spectra of fungal pathogens has not been reported so far, leaving this possibility an open issue. In addition, the development of new analytical algorithms, automation of procedures, and optimization of assays are expected to expand and refine the clinical applications of MALDI-TOF MS technology to AFST in the near future.

## Author Contributions

WF and AL contributed to the conception and design of the study. WF wrote the first draft of the manuscript. WF, AT, EG, and AL contributed to manuscript revision and read and approved the submitted version.

## Conflict of Interest Statement

The authors declare that the research was conducted in the absence of any commercial or financial relationships that could be construed as a potential conflict of interest.
